# Editorial: Tumor microenvironment and personalized therapy of gastrointestinal cancer Ⅱ

**DOI:** 10.3389/fmed.2026.1978110

**Published:** 2026-09-08

**Authors:** Xinxin Wang, Jianming James Tang, Shuang Yang, Shuo Li

**Affiliations:** 1 Senior Department of General Surgery, the First Medical Center of Chinese PLA General Hospital, Beijing, China; 2 School of Medicine, Nankai University, Tianjin, China; 3 Department of Medicine/Division of Infectious Diseases, University of Alabama at Birmingham (UAB) Schools of Medicine, Birmingham, AL, United States

**Keywords:** gastrointestinal cancer, personalized management, prognosis, risk stratification, tumor microenvironment

Gastrointestinal cancers remain challenging malignancies because of their substantial biological and clinical heterogeneity and poor outcomes despite advances in diagnosis and treatment. Increasingly, cancer is understood not only through tumor-intrinsic features but also through interactions with the local tumor microenvironment and systemic host status. As therapeutic options expand, identifying clinically meaningful sources of heterogeneity for risk stratification and individualized management has become increasingly important.

This Research Topic brings together nine articles spanning gastric, colorectal, rectal, and esophageal cancers, addressing this challenge from complementary clinical and translational perspectives. The contributions highlight three interconnected dimensions of individualized cancer care: the role of systemic inflammatory, nutritional, and body-composition parameters in perioperative and postoperative risk assessment; the spatial and temporal heterogeneity of local tumor characteristics, including immune contexture and residual disease patterns; and the application of refined prognostic stratification to guide postoperative management, long-term surveillance, and treatment of rare or atypical disease presentations.

Collectively, these studies emphasize that effective personalization requires consideration of tumor biology, host systemic status, local biological context, and the evolving clinical course. This multidimensional perspective may facilitate more precise risk assessment and individualized management across gastrointestinal malignancies.

Bai et al. conducted a retrospective cohort study to develop and validate a preoperative nomogram for predicting postoperative complications in patients undergoing radical gastrectomy for gastric cancer. By integrating clinical N stage, carcinoembryonic antigen level, sarcopenia, and CT-assessed serosal invasion, the model demonstrated good discriminatory ability in both the training and validation cohorts. These findings highlight the value of incorporating systemic and body-composition parameters into preoperative risk assessment, providing a practical tool for individualized perioperative management.

Chen et al. investigated the predictive value of the postoperative C-reactive protein-to-albumin ratio (CAR) for severe complications following radical gastrectomy in patients with gastric cancer. The findings showed that patients who developed severe complications had significantly higher CRP and CAR levels and lower albumin levels on postoperative day 3 (POD 3). A POD 3 CAR ≥2.85 was associated with an increased risk of both mild and severe complications and prolonged postoperative hospitalization, with an AUC of 0.769 for predicting severe complications. These findings suggest that POD 3 CAR could be a practical biomarker for early postoperative risk stratification.

Wang et al. evaluated the prognostic significance of the pan-immune-inflammation value (PIV) in patients with pT2-4 gastric cancer using a retrospective cohort. Compared with other peripheral blood-based inflammatory indices, PIV showed a stronger ability to discriminate survival outcomes. Elevated PIV was associated with larger tumor size, advanced TNM stage, and Helicobacter pylori infection, while patients with high PIV levels experienced significantly shorter progression-free and overall survival, particularly in stage III–IV disease. Multivariate analysis further confirmed PIV as an independent prognostic factor, highlighting its potential utility as an accessible indicator of tumor burden and systemic inflammatory status.

Jin et al. compared the prognostic performance of the C-reactive protein-albumin-lymphocyte (CALLY) index with conventional inflammatory markers in 152 patients undergoing radical resection for colorectal cancer. The CALLY index demonstrated superior discriminatory ability for survival outcomes, with a higher AUC than the neutrophil-to-lymphocyte, lymphocyte-to-monocyte, and platelet-to-lymphocyte ratios. Patients with higher CALLY values had markedly better 5-year overall survival, and multivariate analysis identified CALLY as an independent prognostic factor. These findings indicate that CALLY, by integrating systemic inflammation, nutritional status, and immune function, may provide a more comprehensive approach to postoperative risk stratification in colorectal cancer.

Wang et al. examined whether preoperative biopsy specimens accurately reflect the immune microenvironment observed in resected gastric and colorectal adenocarcinomas. Paired samples from 38 gastric and 40 colorectal cancer patients revealed substantially greater CD4+ and CD8+ T cell infiltration and PD-L1 expression in gastric resection specimens than in corresponding biopsies, while CD8+ T cell density also increased in colorectal resection samples. Correlations with tumor markers and clinicopathological features further highlighted differences between cancer types. Notably, biopsy-based immune parameters showed limited predictive accuracy for surgical specimens (particularly in colorectal cancer), underscoring the need to consider tumor-specific heterogeneity when evaluating immune biomarkers for treatment decisions.

Wang et al. developed a pathological subtyping strategy to address the heterogeneous prognosis of esophageal squamous cell carcinoma patients with residual disease after neoadjuvant chemoimmunotherapy. Among 176 non-pathological complete responders, patients were classified according to the location of residual disease into T+N+, T+N0, and T0N+ subtypes. This approach effectively differentiated survival outcomes, with T+N+ patients having the poorest prognosis, whereas T0N+ patients showed outcomes comparable to those achieving pathological complete response. Notably, adjuvant immunotherapy was associated with improved survival specifically in the T+N0 subgroup. Prognostic nomograms incorporating pathological subtype and clinicopathological factors further enabled individualized risk assessment.

Liu et al. investigated the prognostic significance of postoperative recurrence patterns in patients with locally advanced rectal cancer treated with neoadjuvant chemoradiotherapy and surgery. Patients who developed postoperative recurrence were categorized according to their initial recurrence patterns as isolated lung metastasis, isolated liver metastasis, or complex recurrence. The initial recurrence pattern was independently associated with post-recurrence survival, with isolated lung metastasis associated with the most favorable outcomes and complex recurrence with the poorest prognosis. In addition, pathological tumor deposit positivity and higher ypTNM stage were identified as adverse factors for recurrence-free survival. Recurrence within 2 years after surgery was also associated with significantly worse subsequent overall survival. These findings highlight the value of recurrence patterns in postoperative risk stratification and individualized surveillance.

Liao et al. reported a rare case of solitary peroneus longus muscle metastasis following radical resection of advanced rectal cancer (T3cN2bM0). The painful metastatic lesion developed only three months after surgery and was confirmed by PET/CT and histopathology. Combined surgical resection and systemic chemotherapy achieved favorable disease control, with no recurrence or further metastasis during 30 months of follow-up, highlighting the potential value of individualized multimodal treatment.

Wang et al. reported a rare case of malignant transformation of a small gastric hyperplastic polyp in a patient with autoimmune gastritis. Endoscopic resection revealed high-grade dysplasia and intramucosal adenocarcinoma. The case highlights the potential association between hypergastrinemia and gastric carcinogenesis in Helicobacter pylori-negative autoimmune gastritis and supports endoscopic resection for early malignant transformation.

